# Autophagy at the postsynapse begins with Rab11 and does not end with dendritic spine pruning

**DOI:** 10.1080/27694127.2024.2346064

**Published:** 2024-05-01

**Authors:** Aleksandra Janusz-Kaminska, Jacek Jaworski

**Affiliations:** a Department of Cell Biology, Emory University School of Medicine, Atlanta, GA, USA; b Laboratory of Molecular and Cellular Neurobiology, International Institute of Molecular and Cell Biology in Warsaw, Warsaw, Poland

**Keywords:** Atg9A, Rab11, mTOR, autophagy, neurons, synaptic plasticity, dendritic spines

## Abstract

Neurons are highly differentiated and compartmentalized cells that conduct cellular processes in a spatiotemporally regulated manner. Autophagy in neurons occurs locally under stimulation and contributes to synaptic plasticity. Little is known about the initial steps leading to autophagy upon neuronal stimulation and the role of autophagic compartments at the postsynaptic part of the synapse. Here, we summarize our recent manuscript on Rab11 role in autophagy initiation in the dendritic spines. We showed that Rab11 maintains in the dendritic spines Atg9A and is necessary for LC3+ vesicles to emerge at the postsynapse. We hypothesize that autophagosomes arise due to an interplay between NMDA receptor stimulation and local mTOR kinase activity. We suggest that autophagosomes are not, in fact, responsible for dendritic spine pruning.

Recent studies demonstrated the involvement of autophagy in neurodevelopment and synaptic transmission. Under baseline conditions, autophagosomes are few in the neuronal cell body and dendrites. Their number increases upon NMDA receptor (NMDAR) stimulation that causes chemical long-term depression (cLTD), and they degrade postsynaptic scaffold proteins and glutamate receptors. Neurons are highly compartmentalized and for correct neurotransmission, many processes occur under strict spatiotemporal regulation. Dendritic spines, which constitute the postsynaptic part of the synapse, are a functionally and morphologically distinct compartment of the neuron. Until recently, it was unclear if and how autophagosomes form in dendritic spines.

Rab11a is a small guanosine triphosphatase (GTPase) at the surface of recycling endosomes that enter dendritic spines. In nonneuronal cells, Rab11a-positive endomembranes can form a platform for the autophagosome assembly. We hypothesized that Rab11a vesicles play a similar role at the postsynapse [1]. First, we examined dissociated cultures of rat hippocampal neurons. Mature neurons were transfected with EGFP-Rab11a expressing plasmid and then subjected to live imaging. We found that Rab11a endosomes become immobilized in the dendritic spines upon INK128-induced mTOR kinase inhibition. Since decreased mTOR kinase activity prompts autophagy, we suspected that stationary Rab11 vesicles act as autophagy nucleation sites.

We investigated this hypothesis with super-resolution microscopy. As a marker of preautophagosomal structures we used Atg9A, a transmembrane protein transported with the recycling endosomes and necessary for autophagosome formation. We found that Atg9A is abundant in the dendritic spines. Atg9A-positive structures increased in prevalence and colocalized more with Rab11 after INK128 treatment. At the same time, their colocalization with Hook3 and syntaxin 12, proteins associated with motile recycling endosomes, decreased.

Next, we asked if Rab11-Atg9A interaction in the dendritic spine has functional consequences. Using live imaging of hippocampal neurons that overexpressed, wild type or dominant-negative (DN, S25N) EGFP-Rab11a with Scarlet-I-Atg9a we monitored distribution of the latter. Neurons that overexpressed Rab11a DN had substantially less Atg9A in the dendritic spines, indicating that Rab11 activity is needed to maintain Atg9A reservoirs at the postsynapse.

We sought whether Rab11 and Atg9A are locally present in the same protein complex. Since Atg9A does not indicate ongoing autophagy, we also checked for LC3B presence. We performed Rab11 pulldown from mouse synaptoneurosomes, a brain tissue preparation enriched in detached and resealed pre- and postsynaptic vesicles that can partially recapitulate processes at the synapse. We demonstrated that Atg9A and both forms of LC3B, LC3I and digested LCII, coimmunoprecipitated with Rab11. In addition, we treated synaptoneurosomes with INK128 alone or with NMDA, which evoked cLTD in neurons in earlier works. Atg9A exhibited a stronger association with Rab11 upon treatment with both INK128 and NMDA. These results confirmed that Rab11, Atg9A and LC3 coincide in the same protein complex and that autophagosomes could form at the postsynapse.

Thus, we returned to live neurons to observe EGFP-LC3+ vesicle dynamics. We recapitulated the same treatments with either INK128, NMDA cLTD application protocol, or both. We found that INK128 alone did not locate autophagosomes in the dendritic spines. Contrary to earlier findings in fixed cells, NMDA alone did not increase LC3+ vesicle number unless the cells exhibited increased autophagy at baseline. For the neurons with initial low LC3+ vesicle count, it took the application of both treatments for multiple puncta to appear. We observed that LC3+ puncta can arrive at the spine but also emerge *in situ*. We reasoned that while brief inhibition of mTOR kinase kept Rab11 vesicles at the dendritic spines and increased accumulation of Atg9A structures, it did not prompt local autophagy. Instead, it facilitated NMDA-triggered LC3+ puncta appearance at the postsynapse. It would follow that NMDARs and mTOR interactors act synergistically and that the dendritic spine maintains a structure ready to initiate autophagy when needed (Figure 1).

**Figure 1.**
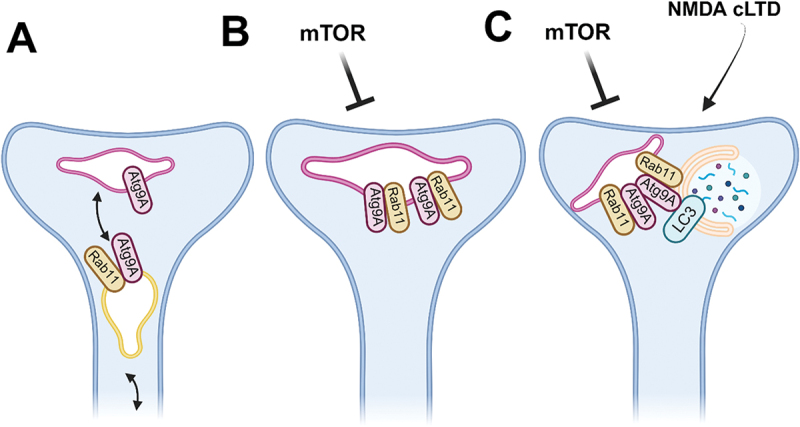
Rab11 participates in autophagosome formation in the dendritic spine (a simplified model). (A). At baseline, Rab11 recycling endosomes supply the postsynaptic site with Atg9A containing endomembranes. (B). When mTOR becomes locally inactive, Rab11 positive endomembranes accumulate with Atg9A positive compartment. (C). Upon NMDAR-driven cLTD, subsequent autophagy-related proteins, including LC3, become recruited to the Rab11-Atg9A compartment and membrane is donated to the newly forming phagophore. Created with BioRender.com.

Inspired by other authors, we wondered whether local autophagy leads to dendritic spine remodeling. We recapitulated live imaging of INK128- and NMDA-treated neurons, then traced the fate of the same spines over ~50 minutes. Although counterintuitive, the same spines where LC3+ vesicles emerged changed less over time and belonged to the pool of larger, stable mushroom spines.

Finally, we repeated the same treatments on neurons overexpressing EGFP-LC3 and Rab11a (wild type or DN), which were additionally pretreated with bafilomycin A to stop the autophagy progression. We found that Rab11a DN variant abolished LC3+ puncta appearance in the dendritic spines under any treatment. This observation proves a causative connection between Rab11a and local autophagy at the postsynapse.

Our research demonstrates the role of Rab11-Atg9A interaction in local autophagy, which is vital for synaptic plasticity. It fills a gap in understanding how the mTOR kinase modulates local, postsynaptic autophagy while it occurs upon neuronal stimulation. Moreover, this process has a more complex function than pruning whole spines. The dendritic spine compartment is a busy hub of protein translation and degradation. Autophagosomes could act as a degradation site and a recycling plant to supply amino acids for translation. Local autophagy could also protect the spines from pruning by rendering them less sensitive to stimulation via receptor degradation. Alternatively, subtle forms of structural plasticity, such as changing the composition of dendritic spine contents, could occur.

## Abbreviations

Atg9, autophagy-related protein 9; EGFP, enhanced green fluorescent protein; LC3, microtubule-associated protein 1 light chain 3; cLTD, chemical long-term depression; mTOR, mammalian target of rapamycin; NMDA, N-methyl-D-aspartate; NMDAR, NMDA receptor.
